# The state of birth asphyxia in Ethiopia: An umbrella review of systematic review and meta-analysis reports, 2020

**DOI:** 10.1016/j.heliyon.2021.e08128

**Published:** 2021-10-05

**Authors:** Wubet Alebachew Bayih, Binyam Minuye Birhane, Demeke Mesfin Belay, Metadel Yibeltal Ayalew, Getachew Yideg Yitbarek, Hailemariam Mekonnen Workie, Dr Misganaw Abie Tassew, Solomon Demis Kebede, Abebaw Yeshambel Alemu, Getnet Gedefaw, Asmamaw Demis, Ermias Sisay Chanie

**Affiliations:** a Debre Tabor University, Ethiopia; b Bahir Dar University, Ethiopia; c Woldia University, Ethiopia

**Keywords:** Birth asphyxia, Umbrella review, Ethiopia

## Abstract

**Background:**

To this date, there are 4 systematic reviews and meta-analyses studies about the burden and associated factors of birth asphyxia in Ethiopia. However, findings of these studies are inconsistent which is difficult to make use of the findings for preventing birth asphyxia in the country. Therefore, umbrella review of these studies is required to pool the inconsistent findings into a single summary estimate that can be easily referred by the information users in Ethiopia.

**Methods:**

PubMed, Science direct, web of science, data bases specific to systematic reviews such as the Cochrane Database of Systematic Reviews and the Database of Abstracts of Reviews of Effects were searched for systematic reviews and meta-analyses (SRM) studies on the magnitude and risk factors of perinatal asphyxia in Ethiopia. The methodological quality of the included studies was assessed using the Assessment of Multiple Systematic Reviews (AMSTAR) tool. The estimates of the included SRM studies on the prevalence and predictors of perinatal asphyxia were pooled and summarized with random-effects meta-analysis models. From checking PROSPERO, this umbrella review wasn't registered.

**Results:**

We included four SRM studies with a total of 49,417 neonates. The summary estimate for prevalence of birth asphyxia was 22.52% (95% CI = 17.01%–28.02%; I^2^ = 0.00). From the umbrella review, the reported factors of statistical significance include: maternal illiteracy [AOR = 1.96; 95% CI: 1.44–2.67], primiparity [AOR = 1.29; 95% CI: 1.03–1.62], antepartum hemorrhage [AOR = 3.43; 95% CI: 1.74–6.77], pregnancy induced hypertension [AOR = 4.35; 95% CI: 2.98–6.36], premature rupture of membrane [AOR = 12.27; 95% CI: 2.41, 62.38], prolonged labor [AOR = 3.18; 95% CI: 2.75, 3.60], meconium-stained amniotic fluid [AOR = 5.94; 95% CI: 4.86, 7.03], instrumental delivery [AOR = 3.39; 95% CI: 2.46, 4.32], non-cephalic presentation [AOR = 3.39; 95% CI: 1.53, 5.26], cord prolapse [AOR = 2.95; 95% CI: 1.64, 5.30], labor induction [AOR = 3.69; 95% CI: 2.26–6.01], cesarean section delivery [AOR = 3.62; 95% CI: 3.36, 3.88], low birth weight [AOR = 6.06; 95% CI: 5.13, 6.98] and prematurity [AOR = 3.94; 95% CI: 3.67, 4.21] at 95% CI.

**Conclusion:**

This umbrella review revealed high burden of birth asphyxia in Ethiopia. The study also indicated significant risk of birth asphyxia among mothers who were unable to read and write, primiparous mothers, those mothers having antepartum hemorrhage, pregnancy induced hypertension, premature rupture of membrane, prolonged labor, meconium-stained amniotic fluid, instrumental delivery, cesarean section delivery, non-cephalic presentation, cord prolapse and labor induction. Moreover, low birth weight and premature neonates were more vulnerable to birth asphyxia compared to their normal birth weight and term counterparts. Therefore, burden of birth asphyxia should be mitigated through special consideration of these risk mothers and neonates during antenatal care, labor and delivery. Mitigation of the problem demands the collaborative efforts of national, regional and local stakeholders of maternal and neonatal health.

## 1 Introduction

Birth asphyxia is defined as “failure to initiate and sustain spontaneous breathing at birth [1, 2]”. It is characterized by marked impairment of exchange of respiratory gases (oxygen and carbon dioxide) resulting in progressive hypoxemia and hypercapnia, accompanied by marked metabolic acidosis [1, 2, 3]. A diagnosis of birth asphyxia can be made when a newborn has fifth minute Apgar score of <7 [2,3]. Besides, a neonate can be labeled as asphyxiated if (a) umbilical cord arterial blood pH < 7; (b) neonatal neurological manifestations (seizures, coma or hypotonia); and (c) multisystem organ dysfunction (cardiovascular, gastrointestinal, hematological, pulmonary or renal system) [4].

Worldwide, 2 to 10 per 1000 term newborns faced perinatal asphyxia [5]. According to Gillam-Krakauer and Gowen, the incidence of birth asphyxia is 2 per 1000 live births in high income countries, but the rate is up to 10 times higher in low income countries where there may be limited access to maternal and neonatal care [6]. As of other evidence, birth asphyxia has an incidence rate of 100–250/1000 live births in low income countries compared to 5–10/1000 live births in the high income world [7, 8]. Besides, a report titled “Birth Asphyxia Complications” estimated the presence of 10 million babies with birth asphyxia at birth [9] which was caused by obstructed labor or acute hemorrhage during birth for which reasons skilled antenatal attendance and emergency obstetric care were best recommended for prevention of the problem [10].

Birth asphyxia has a global significance of causing most of the neonatal deaths [11]. In 2009, more than one million neonatal deaths were attributed to birth asphyxia globally [12, 13]. In 2015, out of 2.68 million neonatal deaths, 637,000 (23.8%) were attributed to birth asphyxia and birth trauma [14]. Besides, in 2019, 2.4 million of the under five deaths were accounted for newborn deaths; and birth asphyxia was blamed to be the leading cause of these deaths [11]. As of 2014, birth asphyxia contributed to less than 0.1% of newborn deaths in high income countries. But, in low income nations, the contribution ranged from 4.6/1000 to 26/1000 live births [15]. In Africa, birth asphyxia accounts for 24.0% of the newborn deaths; and in Sub-Saharan Africa, 280,000 neonatal deaths are accounted for birth asphyxia [16]. More specifically, the incidence of asphyxia in East, Central, and Southern Africa was 22.0% [17].

In addition to the aforementioned mortality burden, birth asphyxia contributes to significant neonatal morbidities due to severe hypoxic-ischemic multi-organ damage, mainly brain damage [18]. These morbidities are of broadly categorized into two types: Immediate and long term. The immediate effects include neonatal hypoxia, hypercarbia, acidosis, hypotension and ischemia whereas the long term morbidities are cerebral palsy, epileptic disorder, motor disorders, developmental delays, speech delays, learning disabilities, mental retardation, hearing impairments, blindness, feeding impairment, and behavioral and emotional disorders [2, 4]. For example, the 2005 World Health Organization report revealed likelihood of developing cerebral palsy, learning difficulties or other disabilities by as many as 1 million survivors of “birth asphyxia” annually [19]. Furthermore, another study [9] showed 233,000 surviving neonates had a moderate or severe disability and another 181,000 had learning problems. Gillam-Krakauer and Gowen also noted that up to 25% of the birth asphyxia survivors are left with permanent neurologic deficits [6]. On the contrary, many asphyctic babies die before they have the chance to develop HIE, attributable to other causes including acquired conditions such as congenital infection, meningitis, hemorrhage, ischemic or hemorrhagic stroke; genetic syndromes or isolated gene conditions; neuro-metabolic disorders particularly where the stress of delivery leads to decompensation and ‘double trouble’ pathologies where a primary pathology leads secondarily to a hypoxic-ischaemic brain injury, like neuromuscular or cardiac disorders. Moreover, some asphyctic babies survive without having HIE [3]. For instance, as of a prospective study at Kilimanjaro Christian Medical College in Tanzania among 201 newborns with birth asphyxia, 14 (6.7%) newborns did not have HIE during the follow up period, and they survived the asphyxia without complication [18]. In Ethiopia, according to the 2019 Mini-Ethiopian Demographic and Health survey report, the neonatal mortality rate is 30/1000 live births [20], and more than 50% of the neonatal deaths occurred within the first day of life [21]. For these deaths, birth asphyxia is the second leading cause of mortality in the country next to prematurity [22, 23, 24, 25, 26]. As of recent evidence, number of neonatal deaths attributable to birth asphyxia and birth trauma declined from 45,965 in 2000 to 28,139 in 2017 in Ethiopia [27]. However, 31.6% of the neonatal mortality in the country is still accounted for birth asphyxia [28], thus contributing to the country's ‘unfinished agenda’ of reducing neonatal mortality rate to as low as 12 per 1000 live births by 2030, which is the key target of Sustainable Development Goal (SDG) [29]. Therefore, it is alarming and warrants an urgent attention of clinicians and health managers to make necessary strategies for preventing birth asphyxia by ensuring quality antenatal, intra-natal and postnatal care at the reach of every woman in the community.

To this date, multiple systematic reviews (SRM) [30, 31, 32, 33] disclosed inconsistent prevalence of birth asphyxia ranging from 21.1% [30] to 24.06% [33] with varying degrees of quality score in Ethiopia. Likewise, there is inconclusive reporting about the effects of different socio-demographic, antenatal, intra-natal and neonatal factors on birth asphyxia. Besides, this umbrella review was in response to the call and recommendation of a prior Ethiopian methodological study [34]. Therefore, the aim of this umbrella review was to summarize the heterogeneous findings of the 4 SRM studies [30, 31, 32, 33] about birth asphyxia into a single comprehensive document where the results of these reviews can be compared and contrasted. To the best of authors’ searching effort, this umbrella review is the first of its kind in addressing birth asphyxia and its predictors in Ethiopia. Hence, evidence from this review will be utilized to guide clinicians and neonatal health policy makers for preventing birth asphyxia in the country, thereby enabling achievement of the SDG target of reducing preventable neonatal mortality to less than 12 deaths per 1000 live births by 2030.

## 2 Methods

This umbrella review was conducted based on the methodology of umbrella review of multiple systematic reviews [35]. It was undertaken through systematic synthesis of the eligible SRM reports on birth asphyxia and its predictors in Ethiopia.

### 2.1 Search strategy

Five international online databases (PubMed, Science direct, web of science, data bases specific to systematic reviews such as the Cochrane Database of Systematic Reviews and the Database of Abstracts of Reviews of Effects) were searched for SRM studies on birth asphyxia in Ethiopia. For accessing relevant data about birth asphyxia, a comprehensive search was conducted through the aforementioned databases using adapted PICO questions. These questions were developed from the following search key words and/or Medical Subject Headings (MeSH) which were combined using the “OR” and “AND” Boolean operators:

a. **Population**: fetus, newborn, and neonate
b. **Outcome**: Fetal distress, hypoxic-ischaemic encephalopathy, postasphyxial encephalopathy, intrauterine asphyxia, intra-partum asphyxia, perinatal asphyxia, perinatal suffocation, neonatal asphyxia, birth asphyxia, postnatal asphyxia, asphyxia neonatorum, suffocation, APGAR score, determinants, predictors, associated factors, correlates, and risk factors,
c. **Study design**: systematic review, meta-analysis of observational studies and
d. **Setting (context)**: Ethiopia

Both published and unpublished studies were searched for this umbrella review. Literature search was conducted from June 28/2020 until August 5/2020. The literature search was performed by two independent researchers, with discrepancies resolved by discussion and consensus. A sample of the literature search strategy, PubMed search strategy, developed using a combination of MeSH terms and free texts is presented as a supplementary file (see Additional file 1).

### 2.2 Eligibility criteria

#### 2.2.1 Inclusion criteria

Publications in the period January 2015–August 2020 were eligible for inclusion. The time restriction was aimed to ensure the findings better reflect or relate to the current neonatal health of the country. The following predefined criteria were considered for a study to be regarded as systematic review or meta-analysis: (a) presented a defined literature search strategy, (b) appraised its included studies using a relevant tool, and (c) followed a standard approach in pooling studies and providing summary estimates.

#### 2.2.2 Exclusion criteria

Studies were excluded due to any of the following reasons: (a) no report on either the prevalence or determinants of birth asphyxia for this study, (b) narrative reviews, editorials, correspondence, abstracts, and methodological studies. Besides, literature reviews that did not have a defined research question, search strategy or defined process of selecting articles were excluded.

### 2.3 Study screening and selection

Searches were downloaded into Endnote version IX and de-duplicated. Then, the screening and selection of studies was conducted in two stages. First, title and abstract screening was conducted. Then, full-text reviewing was done. Through title and abstract screening by two independent researchers (WAB and DMB), studies that mentioned the prevalence and/or determinants of birth asphyxia were selected for full text review. Then, from full-text reviewing, any article classified as potentially eligible by either reviewer was considered as a full text and screened by both reviewers independently. At times of disagreement where a consensus could not be reached between the researchers, a third researcher (BMB) reviewed and resolved the disagreements.

### 2.4 Data extraction

Data from the included SRM studies were extracted using a standardized data abstraction form, developed in excel spreadsheet. For each SRM study, the following data were extracted: (a) identification data (first author's last name and publication year), (b) Review aim (c) prevalence or proportion of birth asphyxia (d) risk factors for birth asphyxia (e) odds ratio or relative risk with 95% confidence intervals for the risk factors of birth asphyxia, (f) number of primary studies included within each SRM study and their respective design type, (g) total number of sample size included, (h) publication bias assessment methods and scores, (i) quality assessment methods and scores, (j) data synthesis methods (random or fixed-effects model), and (k) the authors' main conclusion of the SRM study [Table 1].

**Table 1:** Review characteristics.

Author (year)	Review aim	Search strategy	Included studies	Sample size	Risk of bias	Reported prevalence	Authors' conclusions	AMSTAR Quality
Sendeku et al. (2020) [33]	to assess the pooled prevalence and associated factors of perinatal asphyxia in Ethiopia	Pub Med, HINARI, EMBASE, Google Scholar and African Journals No search start date. No last search date Key search terms not included Limitations described No evidence of hand searching Eligibility criteria: anonymous and editorial reports excluded No evidence of reference checking	Crossectional = 5 Case control = 4	12,249	Clear quality appraisal of the studies has been stated	24.06 (18.11–30.01), I^2^ = 93.5%	Remarkably higher pooled prevalence of perinatal asphyxia determined by prolonged labor, meconium-stained amniotic fluid, instrumental deliveries, and low birth weight	8
Desalew, et al (2020) [31]	To estimate the pooled magnitude and determinants of birth asphyxia in Ethiopia	PubMed, Medline, CINAHL, EMBASE, Google, Google Scholar, and World Health Organization websites. No search start date. Last search date June 2, 2019. Search terms defined. No limitations. Case series and reports were excluded. Both published and unpublished records at any time.	Crossectional = 7 Case control = 4 Cohort = 1	17,147	Clear quality appraisal of the studies has been stated using adapted NOS	22.8 (13–36.8), I^2^ = 83.7%	Very high pooled magnitude of birth asphyxia predicted by maternal education, APH, caesarian section, instrumental delivery, prolonged duration of labor, induction or augmentation, MSAF, and non-cephalic presentation	10
Yoseph Merkeb Alamneh., et al.(2020) [32]	To estimate the pooled prevalence and associated factors of birth asphyxia in Ethiopia	MEDLINE/PubMed, EMBASE, Web of Sciences, Scopus, Crossref, publons, ICMJE, Grey literature databases, Google Scholar, Science Direct, Cochrane library, reference lists of identified studies. No search start date. Last search date November 30, 2019. Search terms defined. Evidence of hand searching from local and national organizations. Evidence of hand searching. Both published and unpublished records at any time. Eligibility criteria: Articles whose full text not accessed after emailing the primary author twice were excluded.	Crossectional = 6 Case control = 4	2,930	The quality of included studies were appraised clearly	22.50 (10.77,34.24); I^2^ = 98.0%	Relatively higher prevalence of birth asphyxia predicted by prolonged labor (>12 h), meconium-stained, assisted vaginal delivery (vacuum or Forceps), C/S delivery, gestational age <37 weeks, non-cephalic presentation, cord prolapse and Premature Rupture of Membrane	8
Assemie et al (2020) [30]	To develop national consensus on pooled prevalence and associated factor of birth asphyxia in Ethiopia	Pub Med/MEDLINE, Google Scholar, Scopus, Science Direct databases, retrieving reference lists of eligible articles and hand searches for grey literature. Search start date January 2019. Last search date April 2019. Search terms defined. Eligibility criteria: Only studies published from April 2014 to April 2019.	Crossectional = 10 Cohort = 5	17,091	Clear evidence of quality assessment for the included primary studies	21.1 (14.08, 28.19); I^2^ = 99.4	High pooled prevalence of perinatal asphyxia significantly influenced by low birth weight, prolonged labor and meconium stained liquor	9

AMSTAR Assessment of Multiple Systematic Reviews.

### 2.5 Risk of bias assessment

All the included studies were critically appraised for validity scoring of their results. To ensure the methodological and evidence quality of the included SRM studies, we used the Assessment of Multiple Systematic Reviews (AMSTAR) tool [36, 37]. The tool consists of 11 questions that measure quality of the approaches used for pooling the empirical studies included in the SRM studies and summarizing their estimates. The quality scoring was done out of 11, with scores 8–11, 4–7, and <3 indicating high, medium, and low qualities, respectively.

### 2.6 Data synthesis

Both narrative (qualitative) and quantitative approaches were used to summarize the estimates of the included SRM studies. When two or more estimates were provided on the prevalence and associated factors of birth asphyxia, we presented the range of the estimates and calculated a summary (pooled) estimate. Choice of the metaanalysis model was guided by the between studies heterogeneity, which was assessed by Higgin's I^2^- Statistics [36]. DerSimonian-Laird random-effects model was used to pool (summarize) prevalence estimates because there was a high level of between-studies heterogeneity [37]. It was not possible to assess publication bias because only four studies were included. A minimum of 10 studies is needed to evaluate publication bias [38, 39]. Stata version 14.0 software was used for the quantitative analyses. A summary list of the predictors of birth asphyxia with their respective odds ratios was prepared.

### 2.7 Ethical consideration

In this study, no study participants’ consent or ethical approval was needed because the study was conducted based on data extracted from SRM studies.

## 3 Results

### 3.1 Literature search findings

**Figure 1:**
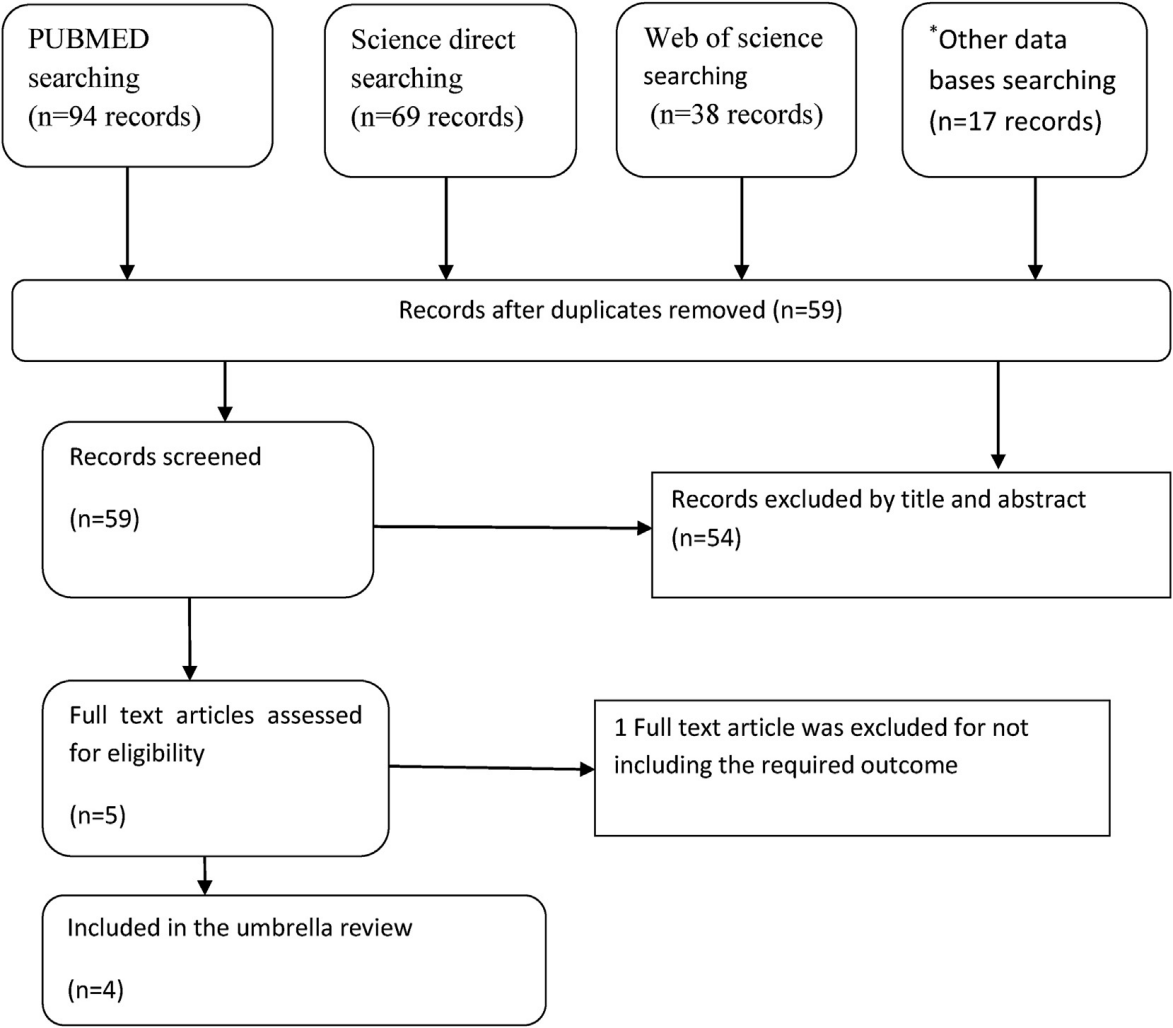
Literature search, screening and selection process (PRISMA flow diagram).

The database search provided a total of 218 articles, of which only 59 articles remained after duplicates became removed. Then, 54 of the 59 articles were excluded by title and abstract screening for not being topics of SRM studies because the objective of this study was to include only SRM studies on birth asphyxia. After full text review of the rest 5 articles, one SRM study was excluded because it didn't consider the required outcome. Thus, a total of 4 SRM studies [30, 31, 32, 33] were included in the current umbrella review. The study selection and screening process is shown in Figure 1.

### 3.2 Characteristics of the included review studies

All the SRM studies [30, 31, 32, 33] included in this umbrella review were based on observational primary studies (6 cohort, 12 case-control, and 28 cross-sectional studies). They included a total of 46 studies, providing a total sample of 49,417 neonates. The number of primary studies per SRM ranged from 9 (lowest) [33] to 15 (highest) [30]. The sample size per meta-analysis ranged from 2,930 (lowest) [32] to 17,147 (highest) [31]. The 3 SRM studies [31, 32, 33] were published in 2020 and addressed both the prevalence and determinants of birth asphyxia. Though published, Alamneh et al [32] cannot be accessed in PubMed because it was published in a journal (acta Scientific MEDICAL SCIENCES) which is not indexed by PubMed. The fourth SRM study, Asemie et al [30], has not been published yet. But, we used to make exhaustive search of both published (even other than PubMed indexed journals) and unpublished sources to reduce publication bias. The given list of databases and other searching techniques under the column of ‘search strategy’ in Table 1 was considered to look for not only the respective SRM study but also each of the primary studies included in the corresponding SRM study. According to the included 4 SRM studies, the reported estimate on the prevalence of birth asphyxia ranged from 21.1% (95%CI: 14.08%, 28.19%), I^2^ = 99.4% [30] to 24.06% (95%CI: 18.1%, 30.01%), I^2^ = 93.5% [33]. General characteristics of the included systematic review and meta-analyses studies are shown in Table 1.

### 3.3 Primary studies

Primary researches within the included 4 SRM studies were mapped to identify if the reviews were based on the same primary evidence. As presented above in Table 1, there were 46 primary studies included within the reviews. However, from critical appraisal of the included 4 SRM studies (column wise) by list of the primary studies (row wise), we found only 23 different primary articles, thus indicating the inclusion of some primary studies by at least two SRM studies. For instance, it is clear that all the four primary studies [42, 44, 45, 53] were included by each of the 4 SRM studies [30, 31, 32, 33]; another four primary studies [40, 43, 46, 47] were considered by each of the three SRM studies [31, 32, 33]; *two primary studies* [48, 51] *belong to each of the 2* SRM studies [30, 31] and one primary study [41] was included by each of the 2 SRM studies [32, 33]. Such an overlap is always expected from any umbrella review; and it has been mentioned among the limitations of the study. On the contrary, 9 primary studies [54, 55, 56, 57, 58, 59, 60, 61, 62] were specific to only Assemie et al [30], 2 primary studies [49, 50] for only Desalew, et al [31], and 1 primary study [52] was included by *Alamneh, et al* [32] *alone indicating that there was no overlapping of data from the aforementioned 12 primary studies* [49, 50, 52, 54, 55, 56, 57, 58, 59, 60, 61, 62] resulting in different prevalence of birth asphyxia among the included 4 SRM studies, which in turn necessitated the conduct of this umbrella review (Table 2).

**Table 2:** Primary studies included in the systematic reviews and meta analyses (SRM).

Review studies	Primary studies																						
	Yohannes K et al [40]	Abebe A et al [41]	Worku N et al [42]	Lisanu W et al [43]	Zelalem J et al [44]	Gdiom G et al [45]	Alemwork D et al [46]	Hagos T et al [47]	Gudayu [48]	Shitemaw *et al* [49]	Worku *et al* [50]	Demisse *et al* [51]	Sebsibie., *et al.* [52]	Ibrahim., *et al.* [53]	Farah et al [54]	Orsido et al. [55]	Weldearegawi et al [56]	Roba et al [57]	Mengesha et al. [58]	Debelew et al [59]	Mehretie et al [60]	Yismaw et al [61]	Demissie et al [62]
Sendeku et al. [33]	∗	#	#	∗	#	#	∗	∗						#									
Desalew, et al [31]	∗		#	∗	#	#	∗	∗	#	#	†	#		#									
Yoseph Merkeb Alamnehet al. [32]	∗	#	#	∗	#	#	∗	∗					#	#									
Assemie et al [30]			#		#	#			#			#		#	†	†	†	∗	†	†	∗	∗	∗

NB: ∗denotes case control studies; **#** crossecrional and **†** cohort studies.

### 3.4 Methodological quality of the included SRM studies

Table 3 shows methodological quality of the included SRM studies, evaluated using the AMSTAR tool for assessment of the methodological quality of SRM studies [36]. The quality scoring was done out of 11 points and ranged from 8 to 10, with a mean score of 9.1 points, indicating an overall moderate quality. The AMSTAR criteria most frequently satisfied across the review studies were those about the priori design, duplicate study selection and data extraction, appropriateness of methods used to combine studies’ findings and disclosure of conflict of interest. The AMSTAR criteria less frequently satisfied were the ones about search comprehensiveness, included and excluded studies provided and scientific quality of the included studies used appropriately in formulating conclusions (Table 3).

**Table 3:** Methodological quality of the included studies based on the AMSTAR tool.

Author, year	Q1	Q2	Q3	Q4	Q5	Q6	Q7	Q8	Q9	Q10	Q11	Total
Sendeku et al. [2020] [33]	Yes	Yes	Yes	No	No	Yes	Yes	No	Yes	Yes	Yes	8
Desalew, et al [2020] [31]	Yes	Yes	Yes	Yes	Yes	Yes	Yes	Yes	Yes	No	Yes	10
Yoseph Merkeb Alamneh., et al. [2020] [32]	Yes	Yes	Yes	Yes	No	No	Yes	No	Yes	Yes	Yes	8
Assemie et al [2020] [30]	Yes	Yes	Yes	Yes	No	Yes	Yes	No	Yes	Yes	Yes	9

AMSTAR Assessment of Multiple Systematic Reviews.

Q1: A priori design; Q2: Duplicate study selection and data extraction; Q3: Search comprehensiveness; Q4: Inclusion of grey literature; Q5: Included and excluded studies provided; Q6: Characteristics of the included studies provided; Q7: Scientific quality of the primary studies assessed and documented; Q8: Scientific quality of included studies used appropriately in formulating conclusions; Q9: Appropriateness of methods used to combine studies' findings; Q10: Likelihood of publication bias was assessed; Q11: Conflict of interest – potential sources of support were clearly acknowledged in both the systematic review and the included studies.

### 3.5 Meta-analysis

#### 3.5.1 Prevalence of birth asphyxia

**Figure 2:**
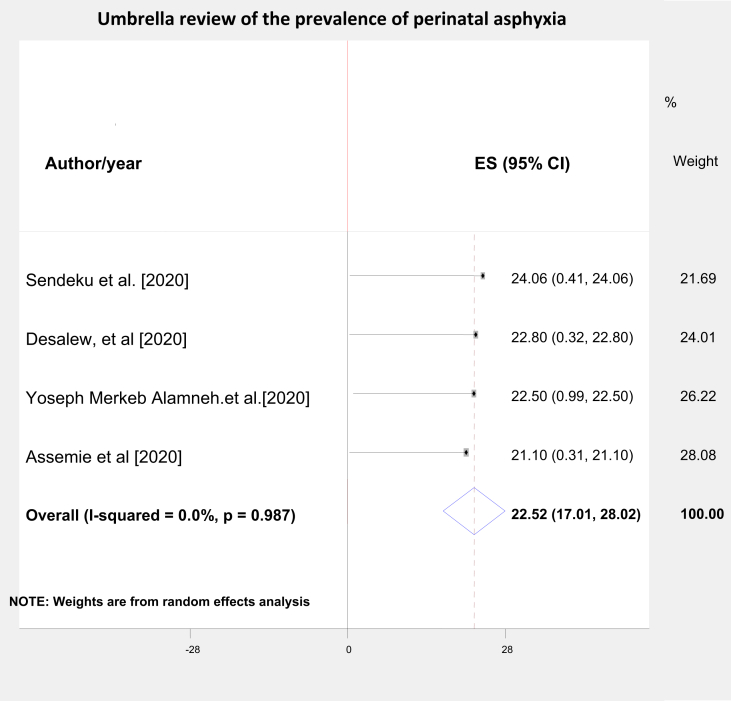
Umbrella review of systematic reviews and met analysis studies on the burden of perinatal asphyxia in Ethiopia.

From umbrella review of the 4 SRM studies [30, 31, 32, 33], the summary (pooled) prevalence of perinatal asphyxia as defined by fifth minute APGAR score below 7 was 22.52% (95% CI = 17.01%–28.02%; I^2^ = 0.00) [Figure 2]. But, the systematic review findings range from 21.1% (95% CI: 14%, 28%) [30] to 24.06% (95% CI: 18.11%, 30.01%) [33] Figure 2.

#### 3.5.2 Risk factors of perinatal asphyxia

Four SRM studies [30, 31, 32, 33] examined a number of factors associated with perinatal asphyxia. The reported factors include maternal educational status, parity, Antepartum Hemorrhage (APH), Pregnancy Induced Hypertension (PIH), Premature Rupture of Membrane (PROM), prolonged labor, Meconium-Stained Amniotic Fluid (MSAF), instrumental delivery, non-cephalic presentation, cord prolapse, induction of labor, cesarean section delivery, low birth weight and prematurity. For this umbrella review, the aforementioned factors are categorized as socio-demographic, antepartum, intrapartum and neonatal factors as detailed in the following subsequent sections.

##### 3.5.2.1 Socio-demographic factors

There was 1 SRM report [31] that showed statistical significance of maternal educational status and parity on the burden of birth asphyxia. According to this report, neonates born to mothers unable to read and write were 2 times (AOR = 1.96, 95% CI: 1.44–2.67) more likely to be asphyxiated as compared to those neonates born to mothers able to read and write. Besides, neonates of primiparous mothers were 1.3 times (AOR = 1.29, 95% CI: 1.03–1.62) more likely to be asphyxiated as compared to neonates of multiparous mothers.

##### 3.5.2.2 Ante-partum factors

One SRM study [31] revealed that neonates born to mothers having APH were 3 folds (AOR = 3.43, 95% CI: 1.74–6.77) likely to be asphyxiated compared to those born to mothers without APH. Furthermore, neonates born to mothers having PIH were 4 times (AOR = 4.35, 95% CI: 2.98–6.36) more likely to be asphyxiated than those neonates born to mothers who didn't have PIH. Another 1 SRM report [32] showed significance of PROM (AOR = 12.27; 95% CI: 2.41, 62.38) in causing perinatal asphyxia.

##### 3.5.2.3 Intra-partum factors

**Figure 3:**
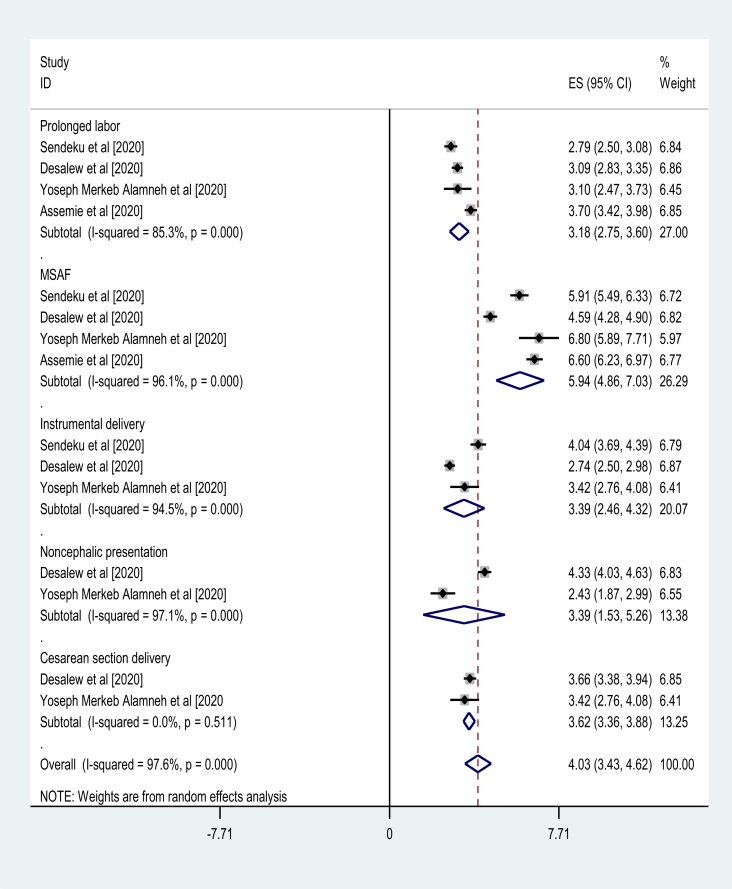
Umbrella review about the pooled effects of intrapartum risk factors on birth asphyxia.

There was 1 SRM report [32] about significance of cord prolapse (AOR = 2.95, 95% CI: 1.64, 5.30) on birth asphyxia. Besides, Desalew et al showed the relevance of labor induction on birth asphyxia (AOR = 3.69, 95% CI: 2.26–6.01). From all SRM reports included [30, 31, 32, 33], the pooled odds of prolonged labor (AOR = 3.18, 95% CI: 2.75, 3.60) and meconium-stained amniotic fluid (AOR = 5.94, 95% CI: 4.86, 7.03) had significant association with birth asphyxia. Three SRM studies [31, 32, 33] also revealed significance of instrumental delivery (AOR = 3.39, 95% CI: 2.46, 4.32) on birth asphyxia. Moreover, 2 SRM reports [31, 32] witnessed importance of the pooled odds of noncephalic presentation (AOR = 3.39, 95% CI: 1.53, 5.26) and cesarean section delivery (AOR = 3.62, 95% CI: 3.36, 3.88) to cause birth asphyxia [Figure 3].

##### 3.5.2.4 Neonatal factors

**Figure 4:**
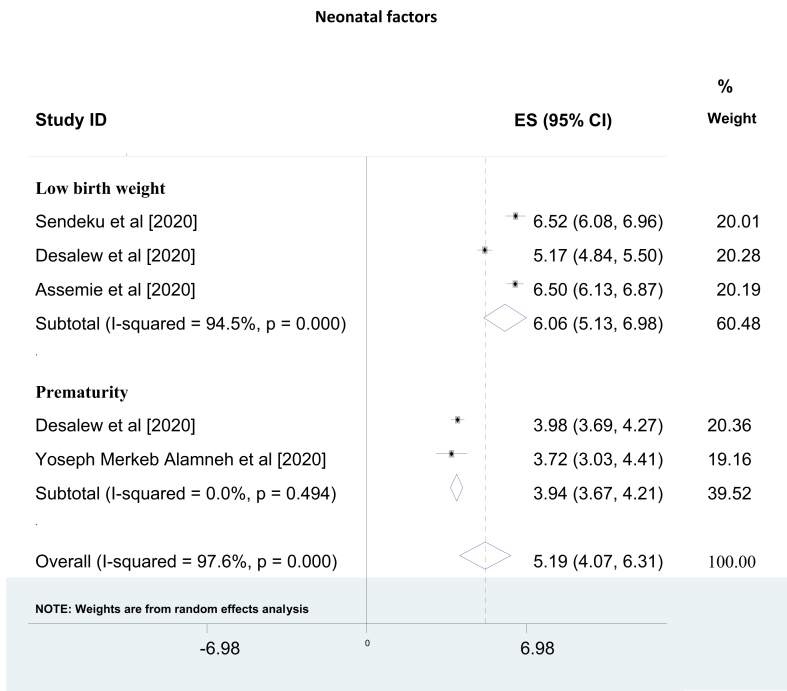
Umbrella review about the pooled effects of neonatal characteristics on birth asphyxia.

The pooled odds of 3 SRM reports [30, 31, 33] about the effect of low birth weight on birth asphyxia showed 6 times higher likelihood of developing birth asphyxia among low birth weight neonates than normal birth weight neonates (AOR = 6.06, 95% CI: 5.13, 6.98). Besides, from 2 SRM studies [31, 32], the pooled odds of having asphyxia among premature neonates were 4 folds higher as compared to term neonates (AOR = 3.94, 95% CI: 3.67, 4.21) [Figure 4].

### 3.6 Conceptual frame work

**Figure 5:**
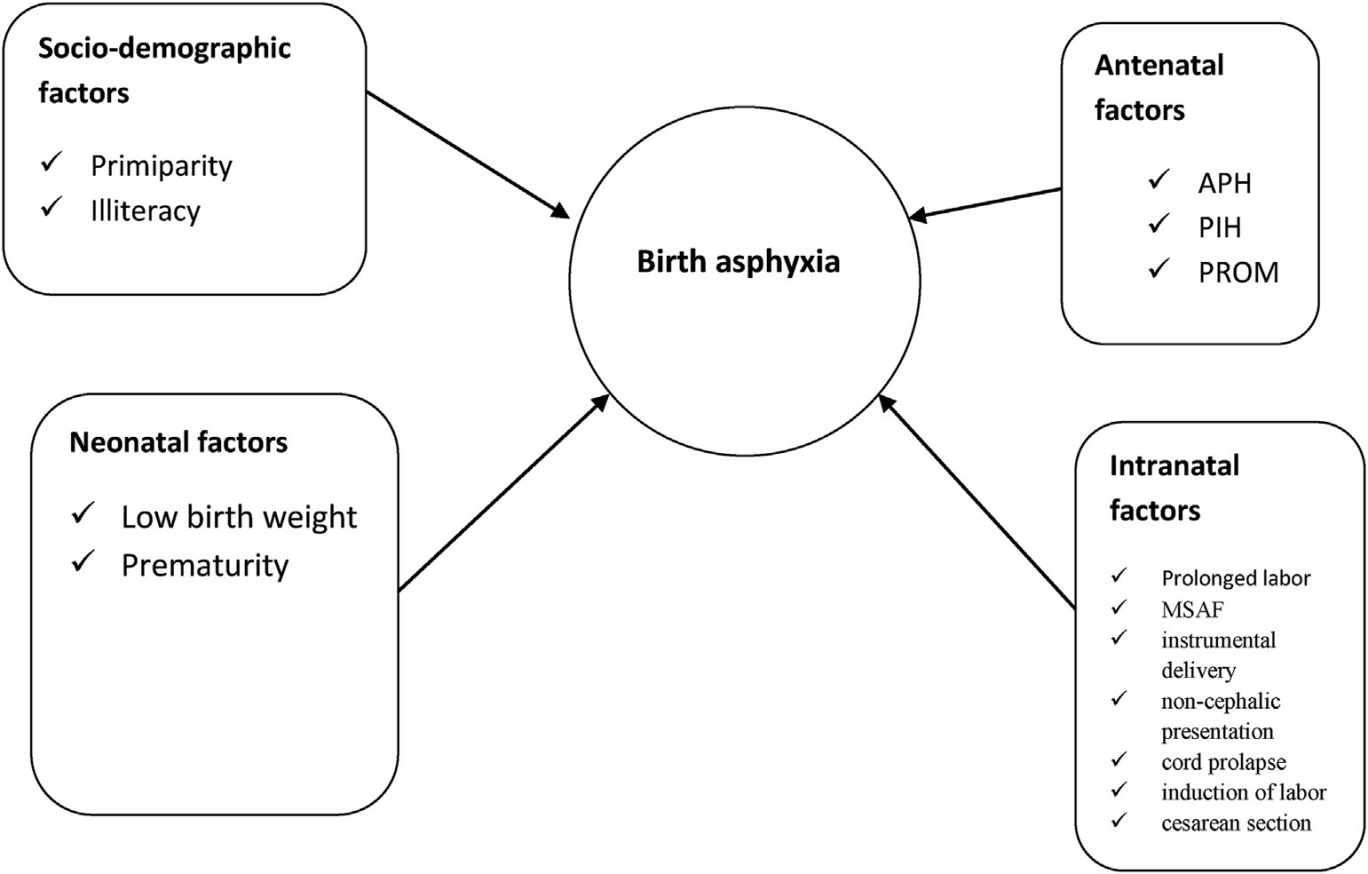
Conceptual framework on the umbrella review of the risk factors for perinatal asphyxia in Ethiopia.

In Ethiopia, umbrella review of the existing SRM studies showed birth asphyxia is a resultant of different factors in the category of maternal socio-demography, antenatal, intra-natal and neonatal components [Figure 5].

## 4 Discussion

To this date, there are four SRM reports about birth asphyxia in Ethiopia. In fact, SRM studies are thought to denote a high level of evidence for decision making in health programs. However, it could be exhausting for the information users when the number of individual reviews increases [35]. Therefore, this umbrella review was conducted to summarize the four SRM studies on birth asphyxia into a single document, and found that birth asphyxia was highly prevalent and a problem of significant public health concern in Ethiopia. Moreover, different factors falling in the category of maternal socio-demography, antenatal period, intra-partum period and neonatal related characteristics were summarized to be of statistical significance in determining the burden of birth asphyxia in the country.

Umbrella review of the included 4 SRM studies on the burden of birth asphyxia in Ethiopia revealed a summary estimate of 22.52% (95% CI: 17.01%, 28.02%) which concurs with its incidence in East Africa 18.0% (95% CI: 11.4%, 26.7%), but higher than in Central African countries 9.1% (95%CI: 2.0%, 16.2%) [63]. The variation in birth asphyxia between Ethiopia and Central African countries may be due to relatively poor maternal socio-demographic characteristics, low antenatal care visits (62%), high home delivery (74%), high prevalence of low birth weight and preterm births in Ethiopia than in Central African countries [9, 11, 14]. Besides, our study involved only Ethiopia while the study in Central Africa included several countries whose prevalence of birth asphyxia was averaged hence relatively lower burden in the region than in Ethiopia. The burden of asphyxia in Ethiopia was; however, lower than in Iran (58.8%) [64] which may be due to difference in case definition; for example, our study was based on only fifth minute APGAR score less than 7 whereas that of the Iranian study used to have a flexible diagnostic criteria of birth asphyxia including: umbilical cord pH < 7 or 5 min Apgar score <6 or 20 min Apgar score less than 7 or multi organs failure in the first 72 h or convulsion in the first 24 h of life.

Birth asphyxia accounts for 24.0% of the neonatal deaths in Africa [16]. Moreover, it comprises 31.6% of the neonatal deaths in Ethiopia [28] indicating its severity in the country. The severity could be due to lack of costly neonatal care of asphyxiated neonates like miracradle, a specialized neonatal bed designed for providing therapeutic hypothermia for neonates suffering from birth asphyxia [2]. As birth asphyxia is a multifactorial condition, concerted efforts should be made in its prevention through mainly skilled emergency obstetrics care [7, 8, 11]. The Ethiopian government is implementing different strategies to reduce birth asphyxia such as accessibility of maternity waiting homes for improving antenatal service usage and institutional delivery rate [20]. But, further work is still needed to reduce neonatal deaths attributable to birth asphyxia. Therefore, obstetric measures that are specific and sensitive to feto-neonatal health should always be emphasized.

Neonates born to mothers unable to read and write were 2 times (AOR = 1.96, 95% CI: 1.44–2.67) more likely to be asphyxiated as compared to those born to mothers able to read and write. Our finding was consistent with studies in southern Nepal [66] and Pakistan [67]. This may be due to the more educated a mother is, the more likely to utilize maternal and neonatal services during pregnancy, labor and postnatal times hence minimizing risk of asphyxia [68]. Thus, unable to read and write mothers should be continuously given health education about optimizing feto-neonatal health during pregnancy at health facilities and even in the community through community health education by encouraging health extension workers.

Besides, neonates of primiparous mothers were 1.3 times (AOR = 1.29, 95% CI: 1.03–1.62) more likely to be asphyxiated as compared to neonates of multiparous mothers and this finding concurred with a Kenyan study [65]. This may be due to primigravidous mothers have a relatively stronger and more vigorous uterine contractions leading to compromised oxygen supply to the fetus hence birth asphyxia [69]. Besides, primigravidous mothers are often subject to induction, which is a known possible risk factor of birth asphyxia from the hyperuterotonic and antidiuretic adverse effects of oxytocin resulting in uterine rupture, water intoxication hence fetoplacental insufficiency and birth asphyxia [70]. Umbilical cord entanglement (nuchal cord) is also more likely among primigravidous mothers hence fetal hypoxia from cord accidents [71, 72]. Thus, primigravida mothors should always be at the forefront of receiving special antenatal and intranatal follow up to prevent or minimize the possible intrapartum related complications as early as possible.

Neonates born to mothers having antepartum derangements (antepartum hemorrhage, pregnancy induced hypertension and premature rupture of membranes) and intrapartum risk factors (prolonged labor, noncephalic presentation and cord prolapse) were more risked for birth asphyxia than their counterparts which accords with findings from Kenya [65], East and Central Africa [63], Pakistan [67] and Iran [64] which could be due to uteroplacental insufficiency hence compromised fetal oxygenation and birth asphyxia. As a result, the existing efforts of health extension workers, health care providers and health policy makers should be pooled to improve the utilization of maternal and child health services during pregnancy and labor, thus optimizing neonatal lives.

Preterm babies were 4 times (AOR = 3.94, 95% CI: 3.67, 4.21) more likely to be asphyxiated than term babies. This study is in line with studies conducted in Jordan [73] and Jakarta [74] which discovered that preterm babies had more risk of developing birth asphyxia than the term counterparts. This may be due to premature infants are more susceptible to ischemia due to incomplete blood brain barrier formation. Moreover, it may be due to the fact that preterm babies face multiple morbidities including organ system immaturity especially lung immaturity causing respiratory failure.

Low birth weight neonates (AOR = 6.06, 95% CI: 5.13, 6.98) had 6 times more likelihood of being asphyxiated as compared to their normal birth weight counterparts Kenya [65], Mulago Hospital, Uganda [75], Dr Soetomo Hospital Surabaya, Indonesia [76], Vali-eAsr Hospital, Tehran-Iran [77], Civil Hospital in Karachi, Pakistan [78], Pattani Hospital, Thailand [79] and Phramongkutklao Hospital, Thailand [80]. This could be explained by the fact that low birth weight babies might be pre-term that they might not have enough surfactant which might lead to suffering from difficulty of breathing and developing difficulty in cardiopulmonary transition and subsequent birth asphyxia [81], [82]. Moreover, small babies have low brown fat tissue which increases their risk of being hypothermic thus increasing the severity of asphyxia. Consequently, low birth weight neonates should be provided with immediate respiratory support, calorie gain and thermal care support to help them adapt the extra uterine environment [1, 2].

### 4.1 Implications of the study

This study was in response to the call and recommendation of a prior Ethiopian methodological study [34] that urged summary evidence on a certain health problem when there is more than one systematic review on that problem. Being the first of its kind in synthesizing the existing SRM reports about birth asphyxia in Ethiopia, this umbrella review has brought a comprehensive summary estimate of the problem. Therefore, this national summary estimate of birth asphyxia and its associated factors can be used by clinicians, policy makers and all other bodies at the stake of optimizing neonatal health in the country.

### 4.2 Strength and limitation

The risk of bias was tried to be minimized through exhaustive searching of multiple databases, and study selection was undertaken by two researchers, with involvement of a third researcher as a tie breaker. Once more, risk of bias of the SRM studies was assessed using the AMSTAR tool. Primary researches within the SRM reports were also mapped to identify the overlap of data among the included SRM studies. Despite the aforementioned strengths, summarizing multiple meta-analyses data that include overlapping primary studies has the potential to overestimate the strength of the findings. Also, usage of the fifth minute Apgar score alone, compared to AAP and ACOG definition of birth asphyxia, does not give a complete picture of measuring birth asphyxia, which might have caused an overestimated prevalence of birth asphyxia in Ethiopia. Therefore, future studies in the country should complement fifth minute Apgar score with immediate newborns’ umblical cord blood pH and bio-chemical results of arterial blood gas analyses (indicative of neonatal hypoxemia and hypercarbia). Moreover, regarding outcome measurement, 2 of the 23 primary articles, namely Necho AW et al [42] and Meshesha AD et al [46] considered the first minute Apgar score <7, unlike the rest 21 primary articles that considered the fifth minute Apgar score <7. This discordance in measuring birth asphyxia might have influenced the generalization and interpretations of our pooled estimate. Most importantly, confounding factors that can affect birth asphyxia were not identified due to the nature of meta-analysis in using aggregated group data, which could have affected the pooled estimate. Because of all the above mentioned reasons, our pooled estimate may not actually represent the national figure of birth asphyxia in Ethiopia. Therefore, we would like to forward our earnest reminder for the readers to be mindful of interpreting and using this finding in the context of both inherent limitations of the included primary studies and the current umbrella analysis.

Overall, since this meta-analysis has systematically identified all the aforementioned limitations, the design of future studies can be substantially improved.

## 5 Conclusions

In this umbrella review, the quality of individual reviews (SRM) were first appraised and then evidence were highlighted and brought together in a single document, providing definitive summaries of birth asphyxia that could be used to inform clinical practice.

From the umbrella review, birth asphyxia is still burdensome in Ethiopia. Besides, maternal illiteracy, primiparity, antepartum hemorrhage, pregnancy induced hypertension, premature rupture of membrane, prolonged labor, meconium-stained amniotic fluid, instrumental delivery, non-cephalic presentation, cord prolapse, labor induction, cesarean section delivery, low birth weight and preterm babies are positively associated with birth asphyxia. All of these factors can be optimized through the collaborative efforts of national, regional and local stakeholders of neonatal health in Ethiopia. As advanced cares like miracradle aren't present for asphyxiated neonates in the country, prevention is unquestionably urged. Thus, health care providers should make exhaustive investment of their efforts for early detection and management of obstetrical deviations during pregnancy, labor and delivery. Most importantly, strict partographic follow ups of feto-maternal health should be made during the intrapartum time supported by different diagnostics (E.g. ultrasound), accompanied with immediate emergency obstetrics and newborn care interventions.

## Declarations

### Author contribution statement

Demeke Mesfin Belay, Metadel Yibeltal Ayalew, Getachew Yideg Yitbarek, Hailemariam Mekonnen Workie, Getnet Gedefaw, Asmamaw Demis, Solomon Demis Kebede, Misganaw Abie Tassew, Abebaw Yeshambel Alemu and Ermias Sisay Chanie: Conceived and designed the experiments; Performed the experiments; Analyzed and interpreted the data; Wrote the paper.

Binyam Minuye Birhane and Wubet Alebachew Bayih: Analyzed and interpreted the data; Wrote the paper.

### Funding statement

This research did not receive any specific grant from funding agencies in the public, commercial, or not-for-profit sectors.

### Data availability statement

Data included in article/supplementary material/referenced in article.

### Declaration of interests statement

The authors declare no conflict of interest.

### Additional information

No additional information is available for this paper.

## Appendix A Supplementary data

Additional file 1 PUBMED search strtegy Additional file 1 PUBMED search strtegy
