# Supplementary material for: The state of birth asphyxia in Ethiopia: An umbrella review of systematic review and meta-analysis reports, 2020
Source: Heliyon. 2021 Oct 5;7(10):e08128. doi: 10.1016/j.heliyon.2021.e08128 (PMC8551510; doi:10.1016/j.heliyon.2021.e08128)
Supplement: Additional file 1 PUBMED search strtegy [file mmc1.docx]

Additional file 1: PubMed Search Strategy

| Search | Query | Search results* |
| --- | --- | --- |
| #1 | Search: **((fetus) OR (newborn)) OR (neonate)** Filters: **Free full text** | 1,560,173 |
| #2 | Search: **(((((((((((((((((Fetal distress) OR (hypoxic-ischaemic encephalopathy)) OR (postasphyxial encephalopathy)) OR (intrauterine asphyxia)) OR (intrapartum asphyxia)) OR (perinatal asphyxia)) OR (perinatal suffocation)) OR (neonatal asphyxia)) OR (birth asphyxia)) OR (postnatal asphyxia)) OR (asphyxia neonatorum)) OR (suffocation)) OR (APGAR score)) OR (determinants)) OR (predictors)) OR (associated factors)) OR (correlates)) OR (risk factors)** Filters: **Free full text** | 3,136 |
| #3 | Search: **(systematic review) OR (metaanalysis)** Filters: **Free full text** | 1,580,713 |
| #4 | Search: **Ethiopia** Filters: **Free full text** | 23,329 |
| #5 | #1 AND #2 AND #3AND#4 | 94 |

**= Date of search: August 5, 2020*
